# Efficacy of Liraglutide in Non-Diabetic Obese Adults: A Systematic Review and Meta-Analysis of Randomized Controlled Trials

**DOI:** 10.3390/jcm11112998

**Published:** 2022-05-25

**Authors:** Joshuan J. Barboza, Mariella R. Huamán, Beatriz Melgar, Carlos Diaz-Arocutipa, German Valenzuela-Rodriguez, Adrian V. Hernandez

**Affiliations:** 1 Unidad de Revisiones Sistemáticas y Meta-Análisis (URSIGET), Vicerrectorado de Investigación, Universidad San Ignacio de Loyola (USIL), Lima 15024, Peru; beamelgar@gmail.com (B.M.); carlosdiaz013@gmail.com (C.D.-A.); german.v.valenzuela@gmail.com (G.V.-R.); adrian.hernandez-diaz@uconn.edu (A.V.H.); 2 Tau Relaped Group, Trujillo 13007, Peru; 3 Facultad de Medicina Humana, Universidad Nacional Mayor de San Marcos, Lima 15001, Peru; mariella.huaman@unmsm.edu.pe; 4 Programa de Atencion Domiciliaria (PADOMI)—EsSalud, Lima, Peru; 5 Health Outcomes, Policy, and Evidence Synthesis (HOPES) Group, University of Connecticut School of Pharmacy, Storrs, CT 06269, USA

**Keywords:** liraglutide, body weight, obesity, hypoglycemia, meta-analysis

## Abstract

Objective: We systematically assessed the efficacy of liraglutide in non-diabetic obese adults. Methods: Six databases were searched up to July 2021 for randomized controlled trials (RCTs) assessing liraglutide versus placebo in obese adults. Primary outcomes were body weight and body mass index (BMI). Secondary outcomes were treatment-emergent adverse events (TEAEs), hypoglycemic episodes, HbA1c, and blood pressure. Effect measures were risk ratio (RR) or mean difference (MD) with their confidence interval (95%CI). Random-effects models and inverse variance meta-analyses were used. Quality of evidence was assessed using GRADE. Results: Twelve RCTs (*n* = 8249) were included. In comparison to placebo, liraglutide reduced body weight (MD −3.35 kg; 95%CI −4.65 to −2.05; *p* < 0.0001), and BMI (MD −1.45 kg/m^2^; 95%CI −1.98 to −0.91; *p* < 0.0001). Liraglutide did not reduce TEAEs (RR 1.08; 95%CI 0.92 to 1.27; *p* = 0.25), and Hb1Ac (MD −0.76%; 95%CI −2.24 to 0.72; *p* = 0.31). Furthermore, it did not increase hypoglycemic episodes (RR 2.01; 95%CI 0.37 to 11.02; *p* = 0.28). Finally, liraglutide reduced systolic blood pressure (MD −3.07 mmHg; 95%CI −3.66 to −2.48; *p* < 0.0001) and diastolic blood pressure (MD −1.01 mmHg; 95%CI −1.55 to −0.47; *p* = 0.0003). Seven RCTs had a high risk of bias. Subgroup analyses by length of treatment and doses had effects similar to the overall analyses. Quality of evidence was low or very low for most outcomes. Conclusions: In non-diabetic obese adults, liraglutide reduced body weight, BMI and blood pressure in comparison to placebo. Adverse events, Hb1Ac levels and hypoglycemic episodes were not different than placebo.

## 1. Introduction

Obesity is a major public health problem, affecting more than 603 million adults across the globe [1]. It may also increase the risk of several diseases, including hypertension, dyslipidemia, type 2 diabetes (T2D), and coronary artery disease. Initial management of obese patients includes a combination of dietary changes, exercise, and behavior modification. Nevertheless, in some cases, this strategy is insufficient and pharmacological treatment is required to achieve and maintain therapeutic goals in terms of weight loss.

Liraglutide is a glucagon-like peptide-1 (GLP-1) agonist and potential weight loss drug [2]. It increases insulin concentrations after eating, prior to the elevation of blood glucose levels [3,4]. Liraglutide is a drug used in obese diabetic patients, which justified the investigation of liraglutide as a treatment for non-diabetic obese people. A study evaluated the efficacy at 12 weeks of low-dose liraglutide on the weight of Taiwanese patients without T2D. Compared to baseline, 5.6% of patients in the liraglutide 1.2 mg group reached weight reduction (*p* < 0.001), whereas in the 0.6 mg group 6.4% reached weight reduction (*p* < 0.001) [5]. However, there was no difference in weight reduction between liraglutide doses (absolute difference 1.2 mg vs. 0.6 mg −0.8%, 95%CI −0.12 to 0.11).

We conducted a systematic review and meta-analysis to evaluate the efficacy and safety of liraglutide in non-diabetic obese adults.

## 2. Materials and Methods

We report the systematic review considering the guidelines of the PRISMA-2020 statement [6]. The protocol of this systematic review has been previously published in PROSPERO (CRD42020172654).

### 2.1. Search of Studies

We searched in different search engines such as Web of Science, Pubmed, Embase, Cochrane Central and Scopus, from inception to 7 October 2021. We performed Mesh terms, Emtree terms and TIAB terms, and we designed different strategies for the selected databases (Search strategy, Supplement). We did not limit our searches by language or year of publication.

### 2.2. Eligibility Criteria

We included studies based on: (i) randomized controlled trials (RCTs), (ii) assessed adults with obesity without diabetes type 1 or 2, (iii) evaluated liraglutide compared with placebo or other drugs. Observational studies (case-control studies or cohort), systematic reviews, case series/reports, abstract of conferences and editorials were excluded.

### 2.3. Selection of Studies

One author (JJB) downloaded all registers, and these were added to Rayyan (https://rayyan.qcri.org/, accessed on 23 March 2022), and duplicate records were removed. Two authors (JBM, MHR) independently reviewed the title and abstract regarding eligibility criteria. Following this step, the full-texts were screened for further evaluation. Differences in selections were addressed with a third author (AVH). Endnote 20 software (Philadelphia, PA, USA) was used for saved registers.

### 2.4. Outcomes

Primary outcomes a were decrease in body mass index (BMI) and body weight loss. Secondary outcomes were treatment-emergent adverse events (TEAEs), hypoglycemic episodes, decrease of HbA1c, and blood pressure. The concepts and definitions of outcomes described by the authors in each of the eligible studies were applied. TEAEs are defined as undesirable or unexpected events, which are not present before medical treatment. It can also be considered as an already present event that worsens in intensity or frequency after the treatment provided [7]. TEAEs included gastro-intestinal disorders (nausea, abdominal pain, vomiting, or diarrhea), nervous system disorders, infections and infestations, and vascular disorders. Types of hypoglycemic events in non-diabetic child and adult were: (a) reactive hypoglycemia (glycemia level <70 mg/dL at the time of symptoms and relief after eating); and (b) fasting hypoglycemia (glycemia <50 mg/dL after an overnight fast, between meals, or after physical activity) [8] Specific types of hypoglycemic events for any hypoglycemia were extracted. Also, author-reported definitions were used.

### 2.5. Data Extraction and Management

Two authors (JBM, MRH) independently extracted the data using a pre-developed standard data extraction form. Disagreements were resolved by consensus, and a third author (AVH) was consulted if needed. Data extracted per study were: name of author, year, type of research, country, number of participants, mean age, initial and maximum dosage of liraglutide, duration of treatment, and primary and secondary outcomes per trial arm with baseline values of continuous outcomes.

### 2.6. Risk of Bias Assessment

The RoB 2.0 tool (Bristol, UK) of the Cochrane Collaboration was used for risk of bias assessment [9]. The risk of bias judged the results as low risk, some concerns, or high risk. RoB 2.0 assessment was performed independently by two authors (JBM and MRH), and discrepancies resolved by discussion or with consultation with a third author (AVH).

### 2.7. Statistical Analyses

For meta-analysis, we performed random effects models and followed the inverse variance method. The Paule-Mandel estimator was used for the assessment of the between-study variance [10]. For continuous outcomes, effects of liraglutide on outcomes were expressed as mean difference (MD) with 95% confidence intervals (95% CIs). For dichotomous outcomes, relative risk (RR) with 95% CIs were assessed. Baseline values of continuous outcomes were adjusted for per trial arm. Statistical heterogeneity of effects among RCTs were evaluated using the I^2^ statistic, with values corresponding to low (<30%), medium (30–60%), and high (>60%) levels of heterogeneity. Subgroup analyses by length of treatment (≤16 versus >16 weeks) and maximum dosage (1.8 versus 3.0 mg/day) for all outcomes were performed. For sensitivity analysis, we changed the model and method of meta-analysis. With regard to the model, we applied fixed-effects, and regarding the methods, the Mantel-Haenzel method for sensitivity analyses for the primary outcomes were performed. We used the *metabin* and *metacont* functions of the meta library of R 3.5.1 (www.r-project.org, 23 March 2022). For publication bias analysis, a funnel plot was used to assess asymmetry that may indicate publication bias.

A summary of findings by GRADE methodology was used to rate the quality of evidence (QoE) per outcome [11]. Risk of bias, indirectness, imprecision, inconsistency, and publication bias were assessed, and QoE were rated as high, moderate, low, and very low. QoE was described in the summary of findings (SoF) tables; GRADEpro GDT was used to create SoF tables (GRADEpro).

## 3. Results

### 3.1. Selection of Studies

**Figure 1:**
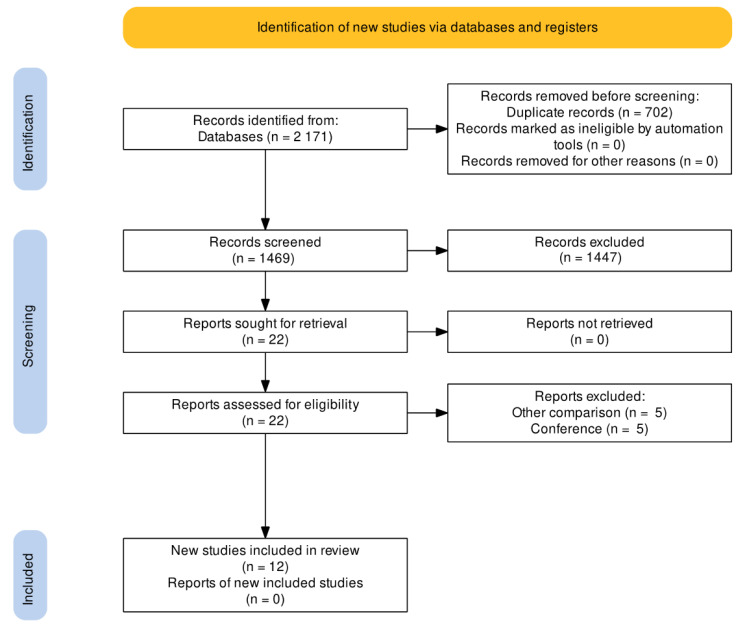
PRISMA flow chart of the study selection process.

After the search, 2171 registers were found in all databases (Figure 1); 702 duplicate registers were deleted. Of 1469 registers, 1447 were excluded by title and abstract. Thus, 22 full-text studies were assessed for eligibility and 10 studies were excluded. Finally, 12 RCTs were included for qualitative and quantitative analyses [4,12,13,14,15,16,17,18,19,20,21,22].

### 3.2. Characteristics of Included Studies

The main characteristics of the included RCTs are summarized in Table 1. A total of 8249 adults treated with liraglutide were evaluated. The mean age was 45.9 ± 5.5 years and 24% of patients were men. Liraglutide was started at 0.6 mg/day with a progressive increase of 0.6 weekly up to a maximum of 1.8 mg/day [13,19,21] and 3.0 mg/day [4,12,14,15,16,17,18,20,22]. The mean duration of treatment was 35.1 ± 19.1 weeks. All studies included body weight loss as primary outcome, and other studies added inflammatory markers [13], glucose tolerance [19], proportion of individuals with T2D [4], and adverse events only [15]. At baseline, the mean Hb1Ac was 5.6% ± 0.09% in the liraglutide arm and 5.6% ± 0.07% in the control arm. Also, the mean BMI was 36.6 ± 2.6 kg/m^2^ in the liraglutide arm and 36.8 ± 2.9 in the control arm.

### 3.3. Risk of Bias

Overall, seven RCTs were scored as high risk of bias [12,13,14,15,20,21,22]. One RCT showed high risk in the randomization process [13]. Three RCTs showed high risk of deviations from intended interventions [13,15,16], and five RCTs showed high risk of missing outcome data [12,13,14,20,22]. The other RCTs showed low or unclear risk of bias (Supplementary Figure S1).

### 3.4. Effect on Primary Outcomes

**Figure 2:**
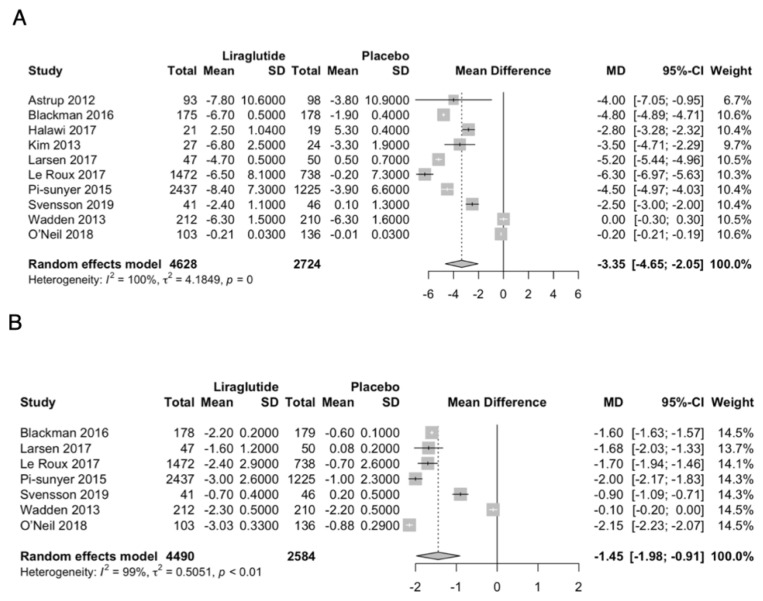
Forest plot of primary outcomes. (**A**): body weight, (**B**): BMI.

In comparison to placebo, liraglutide significantly reduced body weight (MD −3.35 kg; 95% CI −4.65 to −2.05; *p* < 0.0001; I^2^ = 100%; Figure 2A), and reduced BMI (MD −1.45 kg/m^2^; 95% CI −1.98 to −0.91; *p* < 0.0001; I^2^ = 99.5%; Figure 2B).

### 3.5. Effect on Secondary Outcomes

**Figure 3:**
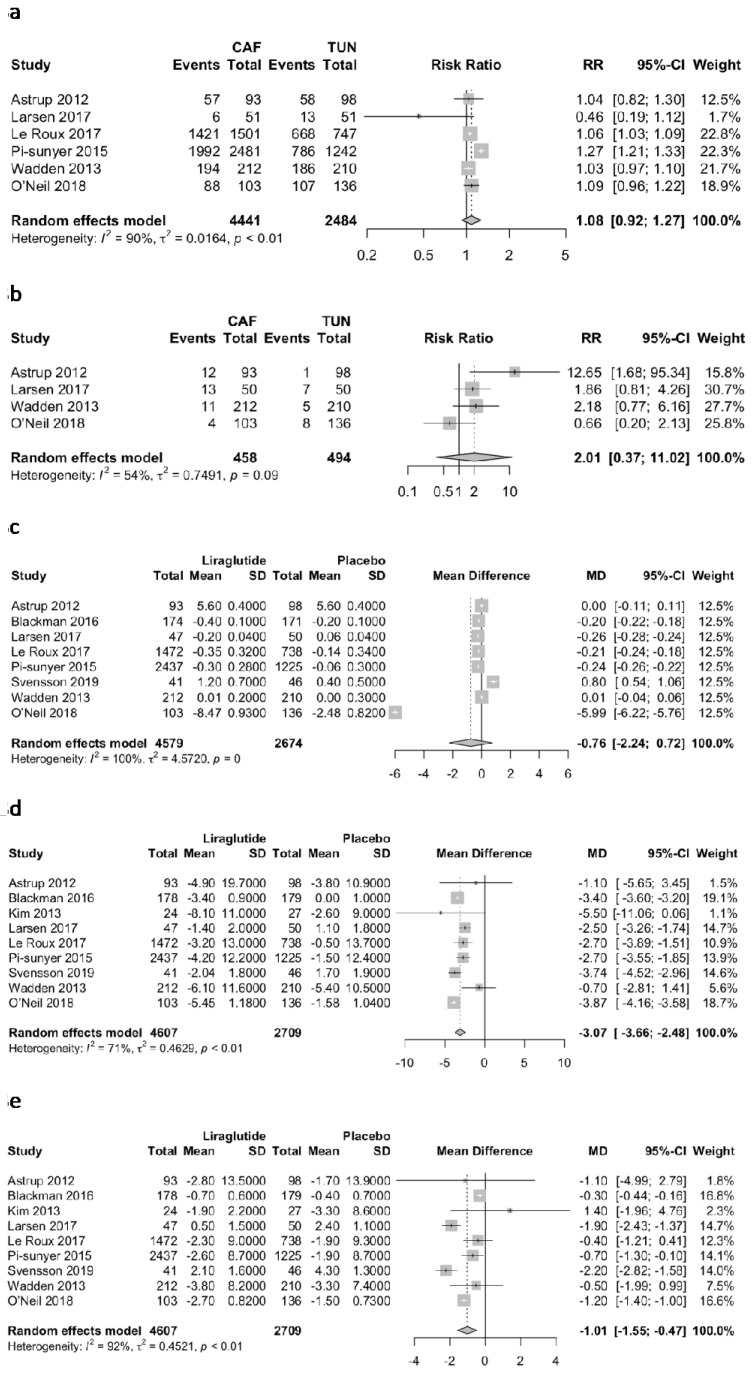
Forest plot of secondary outcomes. (**a**): TEAEs, (**b**): hypoglycemic episodes, (**c**): Hb1Ac, (**d**): Systolic blood pressure, (**e**): Diastolic blood pressure.

Liraglutide did not significantly reduce TEAEs (RR 1.08; 95% CI 0.92 to 1.27; *p* = 0.25; I^2^ = 90.2%; Figure 3a), and did not significantly increase hypoglycemic episodes (RR 2.01; 95% CI 0.37 to 11.02; *p* = 0.28; I^2^ = 54%; Figure 3b) in comparison to placebo. Liraglutide did not reduce Hb1Ac in comparison to placebo (MD −0.76%; 95% CI −2.24 to 0.72; *p* = 0.31; I^2^ = 99.7%; Figure 3c). Finally, liraglutide significantly reduced systolic blood pressure (MD −3.07 mmHg; 95% CI −3.66 to −2.48; *p* = <0.0001; I^2^ = 71%; Figure 3d), and diastolic blood pressure (MD −1.01 mmHg; 95% CI −1.55 to −0.47; *p* = 0.0003; I^2^ = 92.2%; Figure 3e).

### 3.6. Subgroup Analyses

Subgroup analyses by length of treatment and maximum dosage were like the overall analyses for all outcomes (Supplementary Figures S2–S15).

### 3.7. Sensitivity Analyses

Effects on primary outcomes were the same, except for the effects of liraglutide vs. placebo on TEAEs, where liraglutide was associated with higher TEAEs compared to placebo (RR 1.15; 95% CI 1.12 to 1.18; *p* < 0.01) (Supplementary Figures S16 and S17).

### 3.8. Quality of Evidence

QoE was low or very low for most of the primary and secondary outcomes (Supplementary Table S1). In body weight, body mass index, TEAEs, hypoglycemic episodes, Hb1Ac, systolic blood pressure, and diastolic blood pressure, the QoE was very low due to high risk of bias; the heterogeneity among the studies and the imprecision of the effect. In systolic blood pressure, the QoE was low with regard to moderate heterogeneity among the studies. 

### 3.9. Publication Bias

In the graphical test for publication bias, no significant asymmetry indicating high publication bias was observed (Supplementary Figure S18).

## 4. Discussion

### Main Findings

In our systematic review in non-diabetic obese adults, liraglutide reduced body weight, BMI and blood pressure. However, it did not reduce TEAEs episodes or HbA1c, or the risk of hypoglycemic episodes compared with placebo. We also found that liraglutide reduced body weight, BMI, systolic blood pressure, and diastolic blood pressure in comparison to placebo. Subgroup analyses by duration of treatment and maximum dosage were like the main analyses. The risk of bias was high in 30% of the trials. The QoE was low or very low for most of the outcomes.

Liraglutide is a GLP-1 receptor agonist [23]. GLP-1 is known to be a hormone secreted in the intestine, which is activated after food ingestion by enteroendocrine L cells located in the distal jejunum and ileum [24]. It has been found that GLP-1 receptor agonists reduce cardiovascular events in people with T2D and are also a recommended treatment for weight reduction in these patients [25].

GLP-1 receptors are associated with weight loss by attenuating the fall in the anorexigenic hormone leptin that conditions this decrease [3,26]. Based on this, it has been reported that although GLP-1 can increase energy expenditure, its influence on weight is related to decreased energy intake through factors involved with the appetite reward centers of the brain and through local gastrointestinal effects [27].

Some studies have evaluated the efficacy of liraglutide for weight reduction in non-diabetic obese people. For example, a retrospective cohort study [5] evaluated the efficacy of low-dose liraglutide (0.6 vs. 1.2 mg/day) for 12 weeks on body weight among Taiwanese non-diabetic patients. The authors found that among patients in the liraglutide 1.2 mg group, 5.6% reached weight reduction compared to baseline (*p* < 0.001), whereas in the 0.6 mg group 6.4% reached weight reduction (*p* < 0.001); however, no significant differences in weight reduction were found between the two dose groups (absolute difference 1.2 mg vs. 0.6 mg −0.8%, 95%CI −0.12 to 0.11).

In a similar population, a prospective cohort study [28] evaluated the effect of liraglutide on body weight and microvascular function in non-diabetic overweight women with coronary microvascular dysfunction. The authors evaluated the intervention with Liraglutide 3 mg daily for 11 to 13 weeks of treatment, compared to a previous control stage, without treatment, for four to six weeks, and the baseline features. The authors found that a period of 12 weeks of liraglutide 3 mg daily led to a significant weight loss vs. baseline (absolute difference −6.03 kg; 95%CI: −5.22 to −6.84; *p* < 0.001).

A systematic review and Bayesian meta-analysis of RCTs by Khera et al. [29] assessed the effects of different drugs on weight loss and adverse effects in 29,018 patients. The authors included studies that assessed obese (BMI ≥ 30) or overweight (BMI ≥ 27) adults (aged ≥18 years), with or without weight-associated comorbidities. The authors found higher odds of >5% weight loss with the liraglutide group compared to placebo (three studies, 3301 patients, OR 5.09, 95%CI 4.07 to 6.37). A network meta-analysis suggested that phentermine-topiramate, 15 mg/92 mg once daily, was associated with the highest probability of achieving at least 5% weight loss (surface under the cumulative ranking [SUCRA], 0.95), followed by liraglutide (SUCRA, 0.83) and other drugs.

In the 2016 systematic review by Khera et al. [29], the authors did not evaluate the adverse effects or hypoglycemic events. For the liraglutide versus placebo comparison, Khera et al. included 4424 patients, whereas our study included 7236 patients. The Khera et al. study included studies published before 2016. The primary and secondary outcomes were also different, as we included TEAEs, hypoglycemic episodes, body weight, BMI, systolic and diastolic blood pressure and Hb1Ac levels; and they included proportion of patients achieving at least 5% weight loss from baseline, weight loss and adverse events. We used the Cochrane Collaboration RoB 2.0 tool, whereas the study by Khera et al. did not specify the tool used. The study by Khera et al. did not perform subgroup analyses due to a small number of included studies. The inclusion and exclusion criteria between Khera et al. and our study were similar and searched the same databases, but with a different search strategy. In addition, the search and selection of abstracts and full texts was performed independently by two people in the same way as our selection has been carried out. Something in common with the Khera et al. study was the use of the GRADE methodology to evaluate QoE per outcome.

Another systematic review published by Zhang et al. [30], assessed the efficacy and safety of liraglutide in obese, non-diabetic individuals. The authors reported five RCTs involving a total of 4754 patients, and found that mean weight loss (MD = −5.52, 95% CI = −5.93 to −5.11, *p* < 0.00001); loss of more than 5% of body weight (OR = 5.46, 95% CI = 3.57 to 8.34, *p* < 0.00001), and key secondary efficacy end points: SBP decreased (the MD = −2.56, 95% CI = −3.28 to −1.84, *p* < 0.00001). These results are similar to those of our study. However, it is noteworthy that the authors reported a low risk of bias in the trials included in the meta-analysis, whereas our study reported a comprehensive risk of bias analysis, where the majority of trials were found to be at high risk of bias. Another observation is that the authors refer to having used two different models for the meta-analysis, and did not consider the implicit heterogeneity among the studies, and there is no exact distinction about the model applied. Our study, on the other hand, used the random effects model for all meta-analyses under the assumption of heterogeneity and differences between studies.

## 5. Limitations

We have identified several limitations. First, there were differences in the starting and maintenance dose of liraglutide. However, we did not find differences in the weight loss effects between lower or higher liraglutide doses. Second, there was a difference in follow-up time among studies. Most of the included studies had a follow-up time longer than 17 weeks, and our subgroup analyses showed no difference between shorter and longer follow up times. Third, the risk of bias in most studies was high, which may compromise the true effect of most of the outcomes described, as in other studies that applied meta-analysis with included studies and high risk of bias [31,32,33,34]. Finally, in the evaluation of the QoE using GRADE methodology, we found low and very low quality of evidence for most outcomes, which should be considered when interpreting the significant effects that may favor the treatment.

## 6. Conclusions

In non-diabetic obese adults, liraglutide reduced body weight, BMI, and blood pressure in comparison to placebo. TEAEs rates, Hb1Ac and hypoglycemic episodes were not different than placebo. However, the effects in the outcomes may have been compromised due to the true effect related to the high risk of bias in the most studies, and the low or very low level of recommendation in GRADE.

## Figures and Tables

**Table 1:** Baseline characteristics of included randomized controlled trials.

Author	Country	Number of Participants	Age (Mean, SD)	Male (*n*, %)	HbA1c at Baseline (Mean, SD)	BMI kg/m^2^ at Baseline (Mean, SD)	Liraglutide Starting and Maximum Doses	Type of Control	Length of Treatment or Following	Primary Outcomes
Astrup, 2012	Denmark	191	45.9 (10.7)	48 (25%)	LG: 5.6 (0.4); Control: 5.6 (0.4)	NR	Liraglutide 3.0 mg once-daily (increased by 0.6 mg/week)	Placebo	52 weeks	Body weight loss and glycemic parameters
Blackman, 2016	USA	359	48.6 (9.9)	258 (73%)	LG: 5.7 (0.4); Control: 5.6 (0.4)	LG: 38.9 (6.4); Control: 39.4 (7.4)	Liraglutide 3.0 mg once-daily (increased by 0.6 mg/week)	Placebo	32 weeks	Apnea–hypopnea index and Body weight loss
Halawi, 2017	USA	40	37 (29.2)	NR	NR	LG: 37.2 (8.2); Control: 34.6 (6.4)	Liraglutide was administered as recommended by the FDA: initiated at 0.6 mg daily for 1 week, with instructions to increase by 0.6 mg weekly until 3.0 mg was reached (over 4 weeks).	Placebo	16 weeks	Body weight loss
Kim, 2013	USA	51	58 (7)	18 (35%)	NR	LG: 31.9 (2.7); Control: 31.9 (3.5)	The starting dose of medication was 0.6 mg; the dose was titrated by 0.6 mg weekly to a maximum dose of 1.8 mg.	Placebo	14 weeks	Body weight loss and inflammatory markers
Larsen, 2017	Denmark	103	42.1 (10.7)	60 (58%)	LG: 5.6 (0.4); Control: 5.5 (0.4)	LG: 33.7 (5.1); Control: 33.9 (6.6)	The participants followed a fixed uptitration schedule of 0.6 mg per week to a daily dose of 1.8 mg.	Placebo	16 weeks	Glucose tolerance, Body weight loss
Lean, 2014	UK	188	45.9 (10.7)	48 (26%)	NR	LG: 34.8 (2.8); Control: 34.9 (2.8)	Liraglutide doses of 3.0 mg were administered once daily by evening subcutaneous injection, starting with doses of 0.6 mg per day and increasing by weekly increments of 0.6 mg (dose escalation).	Placebo	20 weeks	Adverse events
Le Roux, 2017	USA	2254	NR	540 (24%)	LG: 5.8 (0.3); Control: 5.7 (0.3)	LG: 38.8 (6.4); Control: 39 (6.3)	Start Liraglutide at 0.6 mg with weekly 0.6 mg incremental increases to 3.0 mg.	Placebo	56 weeks	Proportion of individuals with type 2 diabetes, Body weight loss
O’Neil	USA	957	47 (12)	338 (35%)	LG: 5.5 (0.4); Control: 5.5 (0.4)	LG: 38.6 (6.6); Control: 40.1 (7.2)	Liraglutide (3·0 mg) as once-daily subcutaneous injections	Placebo	52 weeks	Body weight loss
Pi-sunyer, 2015	USA	3731	45.2 (12.1)	803 (22%)	LG: 5.6 (0.4); Control: 5.6 (0.4)	LG: 38.3 (6.4); Control: 39.3 (6.3)	Starting at a dose of 0.6 mg with weekly 0.6 mg increments to 3.0 mg	Placebo	56 weeks	Body weight loss
Saxena	USA	56	46 (10.9)	18 (32%)	NR	NR	Liraglutide initiated at a dose of 0.6 mg/day and escalated by 0.6 mg/week up to a maximum of 3.0 mg/day)	Placebo	6 weeks	Change from baseline (CFB) in mean EI (in kcal) during ad libitum lunch meals.
Svensson, 2019	Denmark	97	42.1 (10.7)	60 (62%)	NR	LG: 38.9 (6.4); Control: 39.4 (7.4)	Starting at a dose of 0.6 mg with weekly 0.6-mg increments to 1.8 mg	Placebo	16 weeks	Body weight loss
Wadden, 2013	USA	222	45.9 (11.9)	37 (17%)	LG: 5.6 (0.4); Control: 5.6 (0.4)	LG: 36(5.9); Control: 35.2 (5.9)	Liraglutide 3.0 mg once-daily	Placebo	56 weeks	Body weight loss

SD: Standard deviation; BMI: Body mass index; LG: Liraglutide group; NR: No registered.

## Data Availability

Not applicable.

## Supplementary Materials

The following supporting information can be downloaded at: https://www.mdpi.com/article/10.3390/jcm11112998/s1. Figure S1: Risk of bias assessment of included trials. Figure S2: Subgroup analyses by length of treatment of the effects of Liraglutide vs placebo on TEAEs. Figure S3: Subgroup analyses by doses of the effects of Liraglutide vs placebo on hypoglycemia TEAES. Figure S4: Subgroup analyses by length of treatment of the effects of Liraglutide vs placebo on hypoglycemia episodes. Figure S5: Subgroup analyses by doses of the effects of Liraglutide vs placebo on hypoglycemia episodes. Figure S6: Subgroup analyses by length of treatment of the effects of Liraglutide vs placebo on body weight loss. Figure S7: Subgroup analyses by doses of the effects of Liraglutide vs placebo on body weight loss. Figure S8: Subgroup analyses by length of treatment of the effects of Liraglutide vs placebo on BMI. Figure S9: Subgroup analyses by doses of the effects of Liraglutide vs placebo on BMI. Figure S10: Subgroup analyses by length of treatment of the effects of Liraglutide vs placebo on SBP. Figure S11: Subgroup analyses by doses of the effects of Liraglutide vs placebo on SBP. Figure S12: Subgroup analyses by length of treatment of the effects of Liraglutide vs placebo on DBP. Figure S13: Subgroup analyses by doses of the effects of Liraglutide vs placebo on DBP. Figure S14: Subgroup analyses by length of treatment of the effects of Liraglutide vs placebo on Hb1Ac. Figure S15: Subgroup analyses by doses of the effects of Liraglutide vs placebo on Hb1Ac. Figure S16: Sensitivity analyses of the effects of Liraglutide vs placebo on body weight loss. Figure S17: Sensitivity analyses of the effects of Liraglutide vs placebo on BMI. Figure S18: Publication bias. Table S1: GRADE summary of findings table.
