# Supplementary material for: Efficacy of Liraglutide in Non-Diabetic Obese Adults: A Systematic Review and Meta-Analysis of Randomized Controlled Trials
Source: J Clin Med. 2022 May 25;11(11):2998. doi: 10.3390/jcm11112998 (PMC9181568; doi:10.3390/jcm11112998)

Efficacy and safety of liraglutide in non-diabetic obese adults: a systematic review and meta-analysis

Joshuan J. Barboza, Mariella Huamán-Rivera, Beatriz Melgar, Carlos Diaz-Arocutipa, Adrian V. Hernandez.

Supplementary contents

- **Search strategy for Pubmed, Scopus and Web of Science**
- **Supplemental Figure S1:** Risk of bias assessment of included trials
- **Supplemental Figure S2:** Subgroup analyses by length of treatment of the effects of Liraglutide vs placebo on TEAEs.
- **Supplemental Figure S3:** Subgroup analyses by doses of the effects of Liraglutide vs placebo on hypoglycemia TEAES.
- **Supplemental Figure S4:** Subgroup analyses by length of treatment of the effects of Liraglutide vs placebo on hypoglycemia episodes.
- **Supplemental Figure S5:** Subgroup analyses by doses of the effects of Liraglutide vs placebo on hypoglycemia episodes.
- **Supplemental Figure S6:** Subgroup analyses by length of treatment of the effects of Liraglutide vs placebo on body weight loss.
- **Supplemental Figure S7:** Subgroup analyses by doses of the effects of Liraglutide vs placebo on body weight loss.
- **Supplemental Figure S8:** Subgroup analyses by length of treatment of the effects of Liraglutide vs placebo on BMI.
- **Supplemental Figure S9:** Subgroup analyses by doses of the effects of Liraglutide vs placebo on BMI.
- **Supplemental Figure S10:** Subgroup analyses by length of treatment of the effects of Liraglutide vs placebo on SBP.
- **Supplemental Figure S11:** Subgroup analyses by doses of the effects of Liraglutide vs placebo on SBP.

- **Supplemental Figure S12:** Subgroup analyses by length of treatment of the effects of Liraglutide vs placebo on DBP.
- **Supplemental Figure S13**: Subgroup analyses by doses of the effects of Liraglutide vs placebo on DBP.
- **Supplemental Figure S14:** Subgroup analyses by length of treatment of the effects of Liraglutide vs placebo on Hb1Ac.
- **Supplemental Figure S15:** Subgroup analyses by doses of the effects of Liraglutide vs placebo on Hb1Ac.
- **Supplemental Figure S16:** Sensitivity analyses of the effects of Liraglutide vs placebo on body weight loss.
- **Supplemental Figure S17:** Sensitivity analyses of the effects of Liraglutide vs placebo on BMI.
- **Supplemental Table S1:** GRADE summary of findings table
- **Supplemental Figure S18:** Publication bias

- **Search strategy for Pubmed, Scopus and Web of Science**

PUBMED

1. (Liraglutide OR Victoza OR Saxenda OR “NN 2211” OR “2211, NN” OR “NN2211” OR “NN-2211”)
2. (Obesity OR “Abdominal Obesities” OR “Obesities, Abdominal” OR “Abdominal Obesity” OR “Central Obesity” OR “Central Obesities” OR “Obesities, Central” OR “Obesity, Central” OR “Obesity, Visceral” OR “Visceral Obesity” OR “Obesities, Visceral” OR “Visceral Obesities”)
3. #1 AND #2

SCOPUS

1. TITLE-ABS-KEY(Liraglutide OR Victoza OR Saxenda OR “NN 2211” OR “NN2211” OR “NN-2211”)
2. TITLE-ABS-KEY(Obesity OR “Abdominal Obesities” OR “Abdominal Obesity” OR “Central Obesity” OR “Central Obesities” OR “Visceral Obesity” OR “Visceral Obesities”)
3. #1 AND #2

WOS

1. TS=(Liraglutide OR Victoza OR Saxenda OR “NN 2211” OR “2211, NN” OR “NN2211” OR “NN-2211”)
2. TS=(Obesity OR “Abdominal Obesities” OR “Obesities, Abdominal” OR “Abdominal Obesity” OR “Central Obesity” OR “Central Obesities” OR “Obesities, Central” OR “Obesity, Central” OR “Obesity, Visceral” OR “Visceral Obesity” OR “Obesities, Visceral” OR “Visceral Obesities”)
3. #1 AND #2

Supplemental Figure S1: Risk of bias assessment of included trials

Study ID	Experimental	Comparator	Outcome	Randomization process	Deviations from intended interventions	Missing outcome data	Measurement of the outcome	Selection of the reported result	Overall
Blackman 2016	liraglutide	placebo	Body weight reduction	+	+	?	+	+	!
Halawi 2017	Liraglutide	placebo	Body weight reduction	+	+	+	Ñ	+	+
Kim 2013	Liraglutide	placebo	Body weight reduction	Ñ	Ñ	Ñ	+	?	Ñ
Larsen 2017	Liraglutide	placebo	Body weight reduction	?	+	+	+	+	!
Wadden 2013	Liraglutide	Placebo	Body weight reduction	+	?	Ñ	+	+	Ñ
Le roux 2017	Liraglutide	Placebo	Body weight reduction	?	?	+	+	+	!
Lean 2014	Liraglutide	Placebo	Body weight reduction	?	Ñ	+	+	+	Ñ
Pi-sunyer 2015	Liraglutide	placebo	Body weight reduction	+	Ñ	+	+	+	!
Svensson 2018	Liraglutide	placebo	Body weight reduction	?	?	+	+	+	Ñ
Astrup 2012	Liraglutide	placebo	Body weight reduction	?	+	Ñ	+	+	Ñ
O'Neil 2018	Liraglutide	placebo	Body weight reduction	?	+	Ñ	+	+	Ñ
Saxena 2021	Liraglutide	placebo	Body weight reduction	?	+	Ñ	+	+	Ñ

+ Low risk

? Some concerns

Ñ High risk

Supplemental Figure S2: Subgroup analyses by length of treatment of the effects of Liraglutide vs placebo on TEAEs.

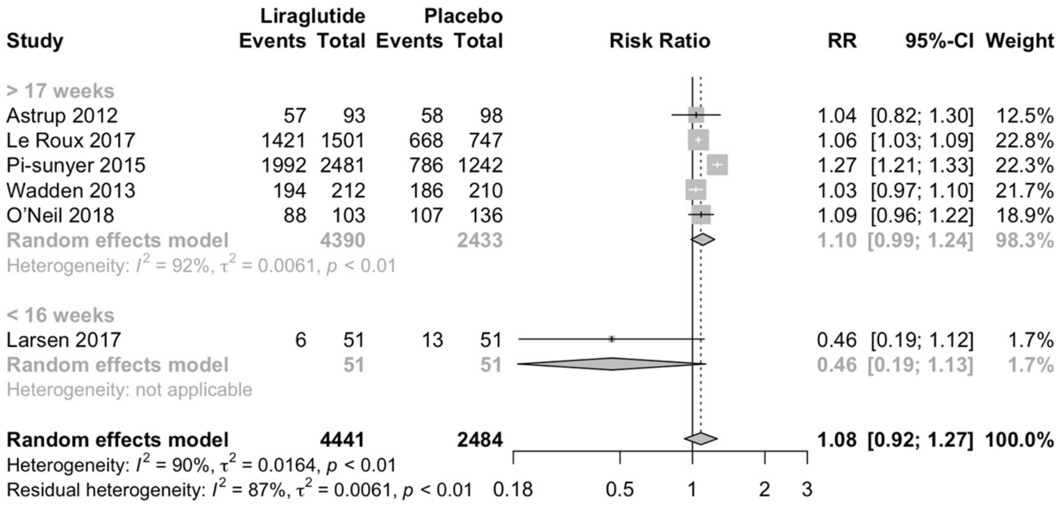

Supplemental Figure S3: Subgroup analyses by doses of the effects of Liraglutide vs placebo on hypoglycemia TEAES.

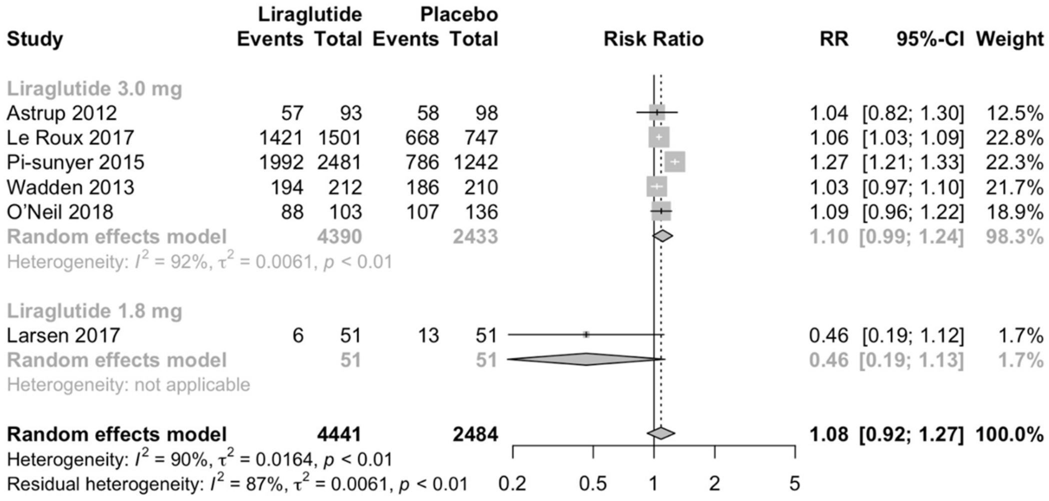

Supplemental Figure S4: Subgroup analyses by weeks of the effects of Liraglutide vs placebo on hypoglycemia episodes.

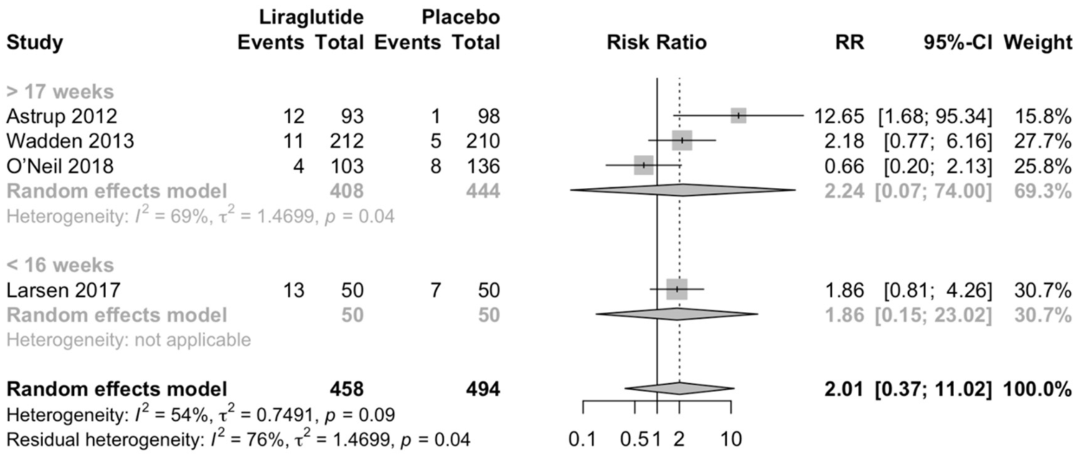

Supplemental Figure S5: Subgroup analyses by doses of the effects of Liraglutide vs placebo on hypoglycemia episodes.

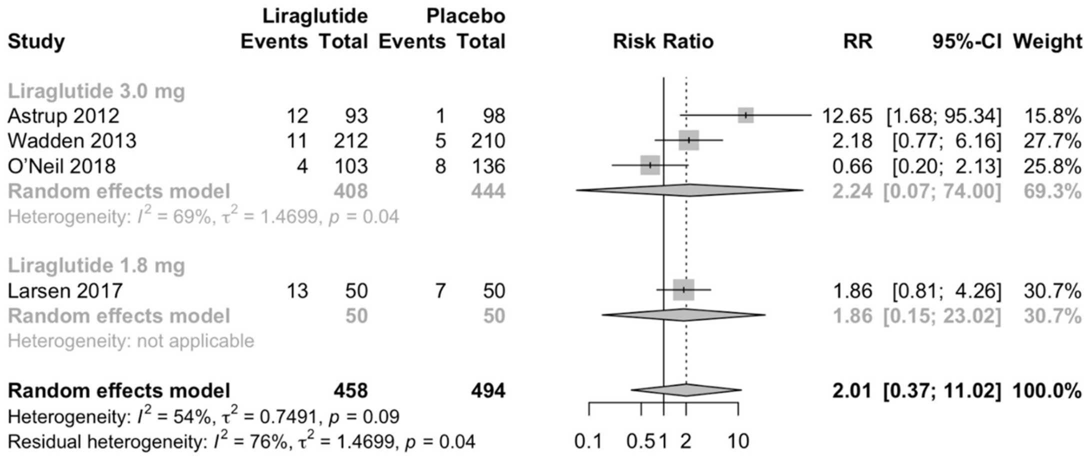

Supplemental Figure S6: Subgroup analyses by weeks of the effects of Liraglutide vs placebo on body weight loss.

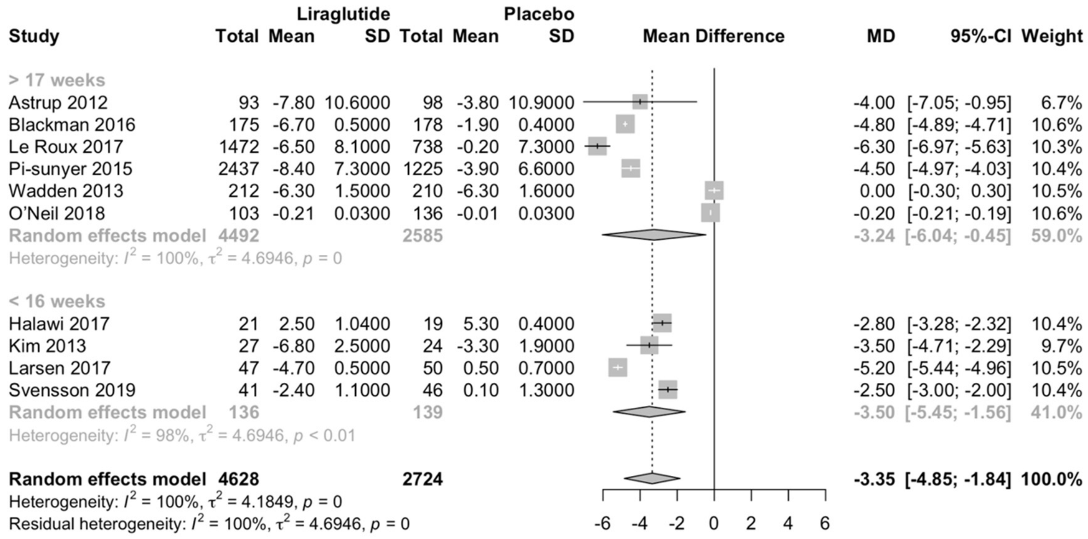

Supplemental Figure S7: Subgroup analyses by doses of the effects of Liraglutide vs placebo on body weight loss.

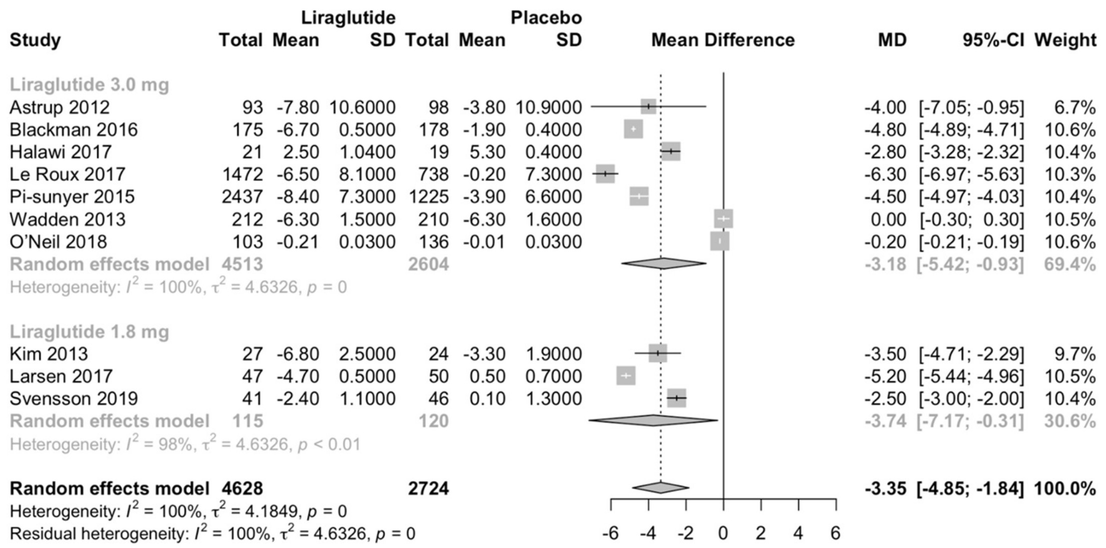

Supplemental Figure S8: Subgroup analyses by weeks of the effects of Liraglutide vs placebo on BMI.

Study	Liraglutide Total	Liraglutide Mean	Liraglutide SD	Placebo Total	Placebo Mean	Placebo SD	MD	95%-CI	Weight
**> 17 weeks**									
Blackman 2016	178	-2.20	0.2000	179	-0.60	0.1000	-1.60	[-1.63; -1.57]	14.5%
Le Roux 2017	1472	-2.40	2.9000	738	-0.70	2.6000	-1.70	[-1.94; -1.46]	14.1%
Pi-sunyer 2015	2437	-3.00	2.6000	1225	-1.00	2.3000	-2.00	[-2.17; -1.83]	14.3%
Wadden 2013	212	-2.30	0.5000	210	-2.20	0.5000	-0.10	[-0.20; 0.00]	14.5%
O'Neil 2018	103	-3.03	0.3300	136	-0.88	0.2900	-2.15	[-2.23; -2.07]	14.5%
**Random effects model**	**4402**			**2488**			**-1.51**	**[-2.53; -0.49]**	**72.0%**
Heterogeneity: $I^2 = 100\%$, $\tau^2 = 0.5927$, $p < 0.01$									
**< 16 weeks**									
Larsen 2017	47	-1.60	1.2000	50	0.08	0.2000	-1.68	[-2.03; -1.33]	13.7%
Svensson 2019	41	-0.70	0.4000	46	0.20	0.5000	-0.90	[-1.09; -0.71]	14.3%
**Random effects model**	**88**			**96**			**-1.28**	**[-6.24; 3.67]**	**28.0%**
Heterogeneity: $I^2 = 93\%$, $\tau^2 = 0.5927$, $p < 0.01$									
**Random effects model**	**4490**			**2584**			**-1.45**	**[-2.11; -0.78]**	**100.0%**
Heterogeneity: $I^2 = 99\%$, $\tau^2 = 0.5051$, $p < 0.01$									
Residual heterogeneity: $I^2 = 100\%$, $\tau^2 = 0.5927$, $p < 0.01$									

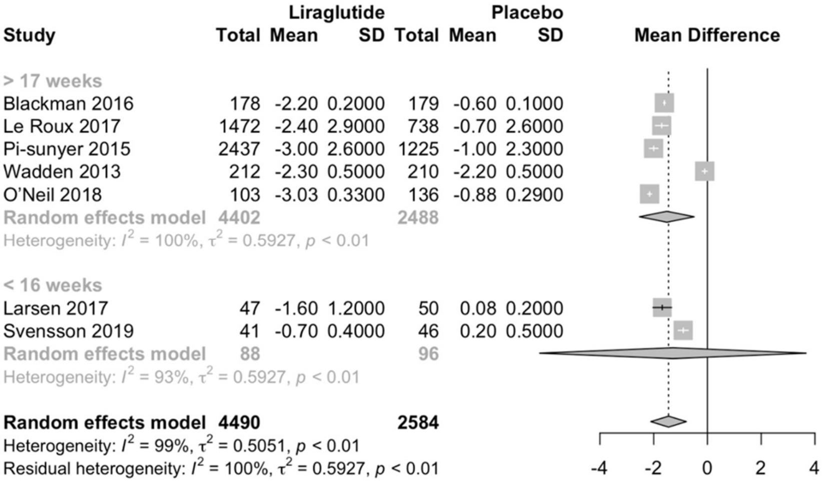

Supplemental Figure S9: Subgroup analyses by doses of the effects of Liraglutide vs placebo on BMI.

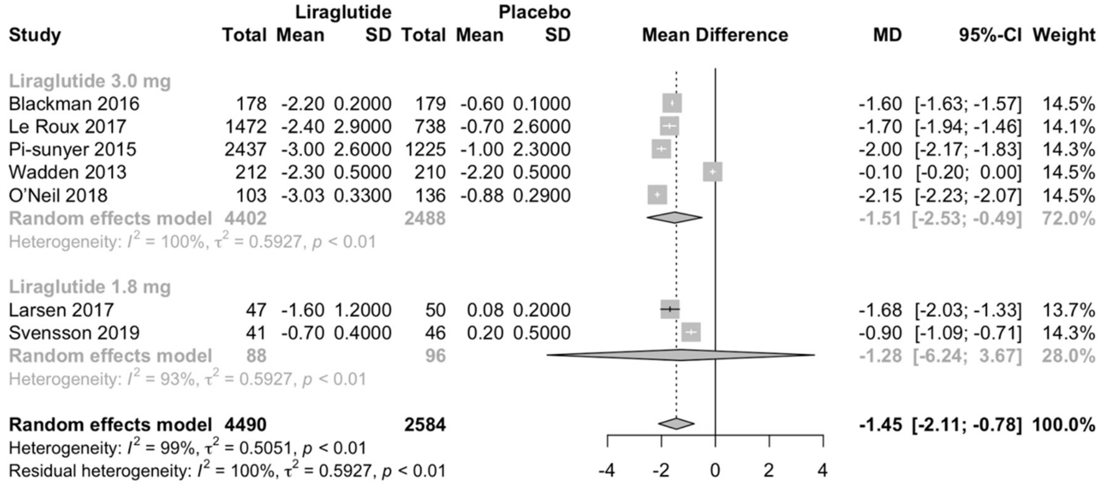

Supplemental Figure S10: Subgroup analyses by weeks of the effects of Liraglutide vs placebo on SBP.

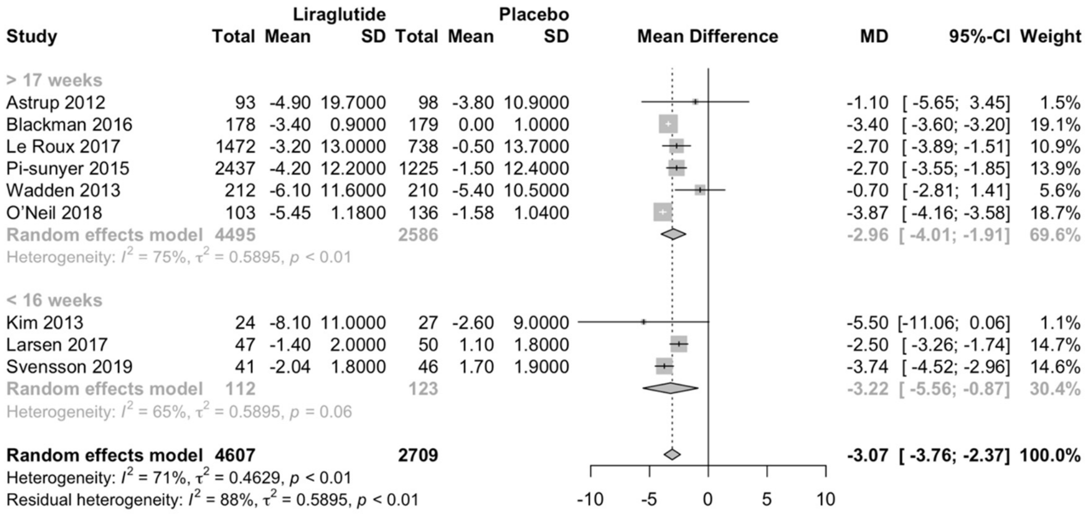

Supplemental Figure S11: Subgroup analyses by doses of the effects of Liraglutide vs placebo on SBP.

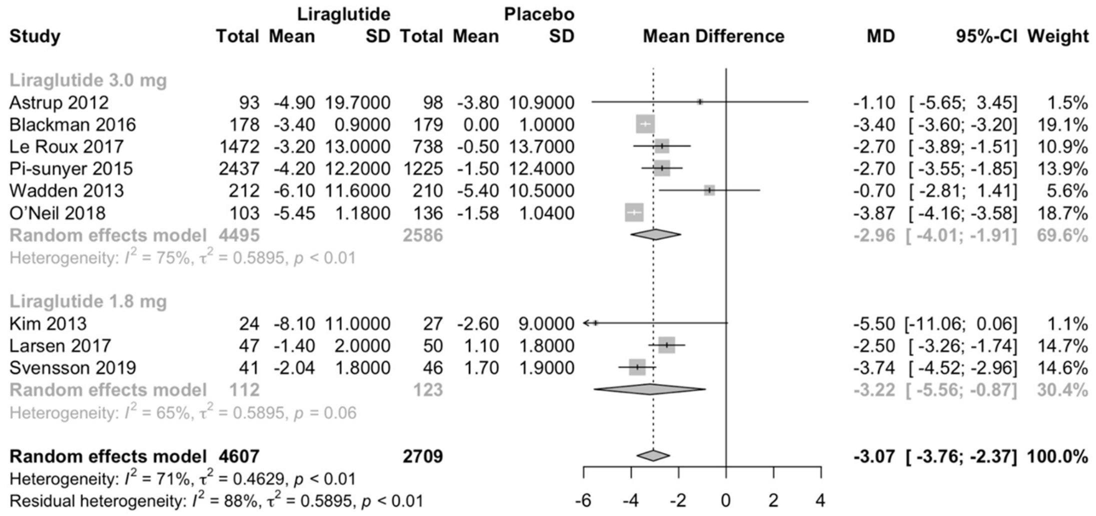

Supplemental Figure S12: Subgroup analyses by weeks of the effects of Liraglutide vs placebo on DBP.

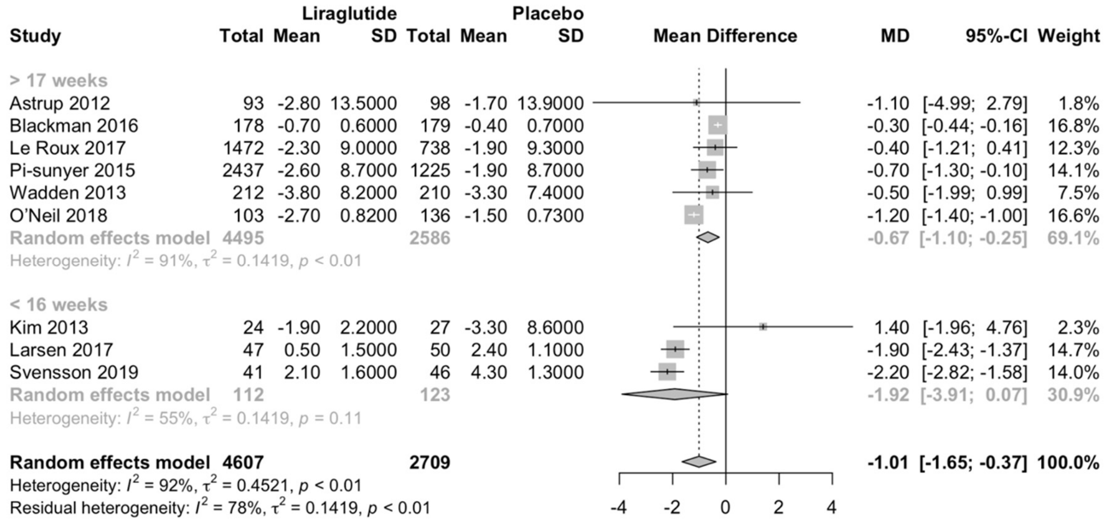

Supplemental Figure S13: Subgroup analyses by doses of the effects of Liraglutide vs placebo on DBP.

Study	Liraglutide Total	Liraglutide Mean	Liraglutide SD	Placebo Total	Placebo Mean	Placebo SD	MD	95%-CI	Weight
**Liraglutide 3.0 mg**									
Astrup 2012	93	-2.80	13.5000	98	-1.70	13.9000	-1.10	[-4.99; 2.79]	1.8%
Blackman 2016	178	-0.70	0.6000	179	-0.40	0.7000	-0.30	[-0.44; -0.16]	16.8%
Le Roux 2017	1472	-2.30	9.0000	738	-1.90	9.3000	-0.40	[-1.21; 0.41]	12.3%
Pi-sunyer 2015	2437	-2.60	8.7000	1225	-1.90	8.7000	-0.70	[-1.30; -0.10]	14.1%
Wadden 2013	212	-3.80	8.2000	210	-3.30	7.4000	-0.50	[-1.99; 0.99]	7.5%
O'Neil 2018	103	-2.70	0.8200	136	-1.50	0.7300	-1.20	[-1.40; -1.00]	16.6%
**Random effects model**	**4495**			**2586**			**-0.67**	**[-1.10; -0.25]**	**69.1%**
Heterogeneity: $I^2 = 91\%$, $\tau^2 = 0.1419$, $p < 0.01$									
**Liraglutide 1.8 mg**									
Kim 2013	24	-1.90	2.2000	27	-3.30	8.6000	1.40	[-1.96; 4.76]	2.3%
Larsen 2017	47	0.50	1.5000	50	2.40	1.1000	-1.90	[-2.43; -1.37]	14.7%
Svensson 2019	41	2.10	1.6000	46	4.30	1.3000	-2.20	[-2.82; -1.58]	14.0%
**Random effects model**	**112**			**123**			**-1.92**	**[-3.91; 0.07]**	**30.9%**
Heterogeneity: $I^2 = 55\%$, $\tau^2 = 0.1419$, $p = 0.11$									
**Random effects model**	**4607**			**2709**			**-1.01**	**[-1.65; -0.37]**	**100.0%**
Heterogeneity: $I^2 = 92\%$, $\tau^2 = 0.4521$, $p < 0.01$									
Residual heterogeneity: $I^2 = 78\%$, $\tau^2 = 0.1419$, $p < 0.01$									

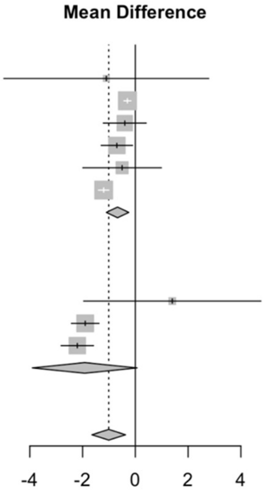

Supplemental Figure S14: Subgroup analyses by weeks of the effects of Liraglutide vs placebo on Hb1Ac.

Study	Liraglutide Total	Liraglutide Mean	Liraglutide SD	Placebo Total	Placebo Mean	Placebo SD	MD	95%-CI	Weight
**> 17 weeks**									
Blackman 2016	178	-2.20	0.2000	179	-0.60	0.1000	-1.60	[-1.63; -1.57]	14.5%
Le Roux 2017	1472	-2.40	2.9000	738	-0.70	2.6000	-1.70	[-1.94; -1.46]	14.1%
Pi-sunyer 2015	2437	-3.00	2.6000	1225	-1.00	2.3000	-2.00	[-2.17; -1.83]	14.3%
Wadden 2013	212	-2.30	0.5000	210	-2.20	0.5000	-0.10	[-0.20; 0.00]	14.5%
O'Neil 2018	103	-3.03	0.3300	136	-0.88	0.2900	-2.15	[-2.23; -2.07]	14.5%
Random effects model	4402			2488			-1.51	[-2.53; -0.49]	72.0%
Heterogeneity: $I^2 = 100\%$, $\tau^2 = 0.5927$, $p < 0.01$									
**< 16 weeks**									
Larsen 2017	47	-1.60	1.2000	50	0.08	0.2000	-1.68	[-2.03; -1.33]	13.7%
Svensson 2019	41	-0.70	0.4000	46	0.20	0.5000	-0.90	[-1.09; -0.71]	14.3%
Random effects model	88			96			-1.28	[-6.24; 3.67]	28.0%
Heterogeneity: $I^2 = 93\%$, $\tau^2 = 0.5927$, $p < 0.01$									
**Random effects model**	**4490**			**2584**			**-1.45**	**[-2.11; -0.78]**	**100.0%**
Heterogeneity: $I^2 = 99\%$, $\tau^2 = 0.5051$, $p < 0.01$									
Residual heterogeneity: $I^2 = 100\%$, $\tau^2 = 0.5927$, $p < 0.01$									

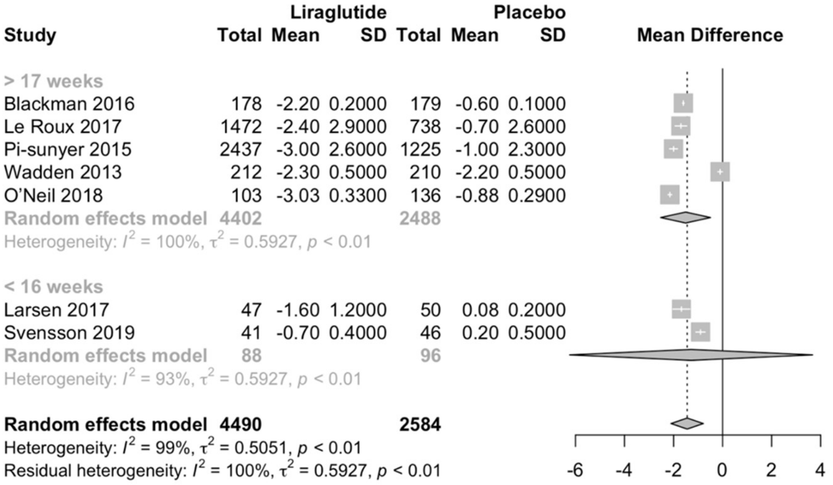

Supplemental Figure S15: Subgroup analyses by doses of the effects of Liraglutide vs placebo on Hb1Ac.

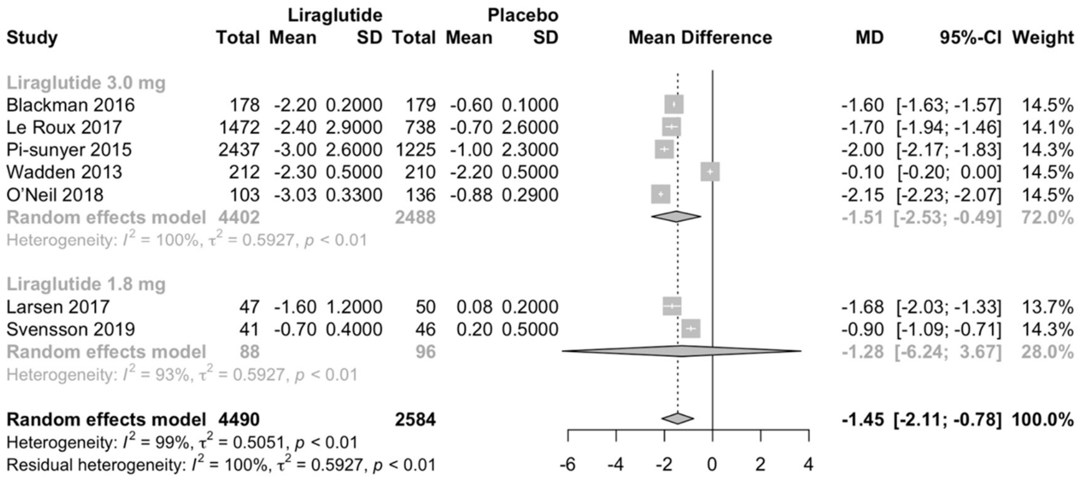

Supplemental Figure S16: Sensitivity analyses of the effects of Liraglutide vs placebo on body weight loss.

	Liraglutide			Placebo					
Study	Total	Mean	SD	Total	Mean	SD	MD	95%-CI	Weight
Astrup 2012	93	-7.80	10.6000	98	-3.80	10.9000	-4.00	[-7.05; -0.95]	0.0%
Blackman 2016	175	-6.70	0.5000	178	-1.90	0.4000	-4.80	[-4.89; -4.71]	0.7%
Halawi 2017	21	2.50	1.0400	19	5.30	0.4000	-2.80	[-3.28; -2.32]	0.0%
Kim 2013	27	-6.80	2.5000	24	-3.30	1.9000	-3.50	[-4.71; -2.29]	0.0%
Larsen 2017	47	-4.70	0.5000	50	0.50	0.7000	-5.20	[-5.44; -4.96]	0.1%
Le Roux 2017	1472	-6.50	8.1000	738	-0.20	7.3000	-6.30	[-6.97; -5.63]	0.0%
Pi-sunyer 2015	2437	-8.40	7.3000	1225	-3.90	6.6000	-4.50	[-4.97; -4.03]	0.0%
Svensson 2019	41	-2.40	1.1000	46	0.10	1.3000	-2.50	[-3.00; -2.00]	0.0%
Wadden 2013	212	-6.30	1.5000	210	-6.30	1.6000	0.00	[-0.30; 0.30]	0.1%
O'Neil 2018	103	-0.21	0.0300	136	-0.01	0.0300	-0.20	[-0.21; -0.19]	99.1%
**Fixed effect model**	**4628**			**2724**			**-0.24**	**[-0.25; -0.23]**	**100.0%**

Heterogeneity: $I^2 = 100\%$, $\tau^2 = 4.1849$, $p = 0$

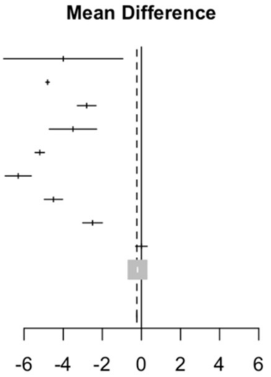

Supplemental Figure S17: Sensitivity analyses of the effects of Liraglutide vs placebo on BMI.

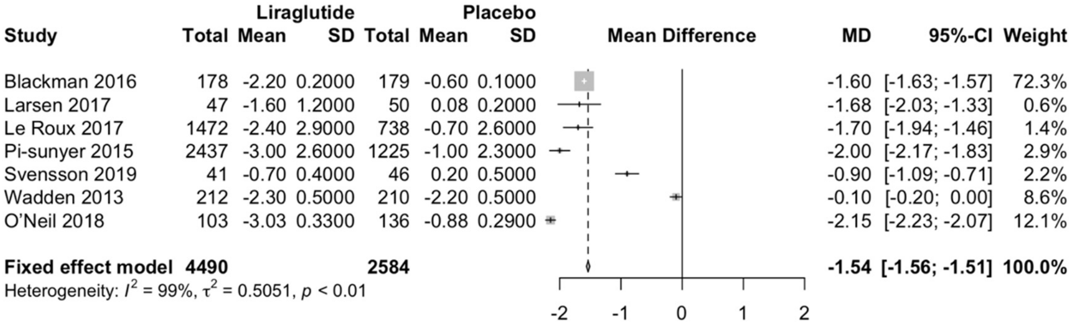

Supplemental Table S1: GRADE Summary of findings of the effect of liraglutide vs. placebo in non-diabetic obese patients

Liraglutide compared to Placebo for body weight loss

Patient or population: body weight loss
Setting:
Intervention: Liraglutide
Comparison: Placebo

Outcomes	№ of participants (studies) Follow-up	Certainty of the evidence (GRADE)	Relative effect (95% CI)	Anticipated absolute effects	
				Risk with Placebo	Risk difference with Liraglutide
TEAEs follow-up: mean 47.2 weeks	6925 (6 RCTs)	⊕◯◯◯ Very low[a,b,c]	**RR 1.08** (0.92 to 1.27)	732 per 1,000	**59 more per 1,000** (59 fewer to 198 more)
Hypoglycemic episodes follow-up: mean 41.3 weeks	952 (4 RCTs)	⊕◯◯◯ Very low[a,d,e]	**RR 2.01** (0.37 to 11.02)	43 per 1,000	**43 more per 1,000** (27 fewer to 426 more)
Body weight follow-up: mean 34.9 weeks	7352 (10 RCTs)	⊕◯◯◯ Very low[a,f,g]	-	The mean body weight was **-1.5** kg	MD **3.35 kg lower** (4.65 lower to 2.05 lower)
Body mass index (BMI) follow-up: mean 38.7 weeks	7074 (7 RCTs)	⊕◯◯◯ Very low[a,f,h]	-	The mean body mass index was **-0.7** kg/m2	MD **1.45 kg/m2 lower** (1.98 lower to 0.91 lower)

Liraglutide compared to Placebo for body weight loss

Patient or population: body weight loss
Setting:
Intervention: Liraglutide
Comparison: Placebo

Outcomes	№ of participants (studies) Follow-up	Certainty of the evidence (GRADE)	Relative effect (95% CI)	Anticipated absolute effects	
				Risk with Placebo	Risk difference with Liraglutide
Systolic blood pressure (SBP) follow-up: mean 37.7 weeks	7316 (9 RCTs)	⊕⊕◯◯ Low[a,h,i]	-	The mean systolic blood pressure was **-1.37** mmHg	MD **3.07 mmHg lower** (3.66 lower to 2.48 lower)
Diastolic blood pressure (DBP) follow-up: mean 37.7 weeks	7316 (9 RCTs)	⊕◯◯◯ Very low[a,h,j]	-	The mean diastolic blood pressure was **-0.7** mmHg	MD **1.01 mmHg lower** (1.55 lower to 0.47 lower)
Hb1Ac (Hb1Ac) follow-up: mean 38 weeks	7253 (8 RCTs)	⊕◯◯◯ Very low[a,k,l]	-	The mean hb1Ac was **0.8** %	MD **0.76 % lower** (2.24 lower to 0.72 higher)
Bilirrubina total (BT) assessed with: MD follow-up: mean 15 days	230 (8 observational studies)	⊕◯◯◯ Very low[a,b]	-	The mean bilirrubina total was **10** mg/dl	MD **2 mg/dl lower** (4 lower to 1 lower)

***The risk in the intervention group** (and its 95% confidence interval) is based on the assumed risk in the comparison group and the **relative effect** of the intervention (and its 95% CI).

CI: confidence interval; **MD:** mean difference; **RR:** risk ratio

Liraglutide compared to Placebo for body weight loss					
**Patient or population:** body weight loss<br>**Setting:**<br>**Intervention:** Liraglutide<br>**Comparison:** Placebo					
**Outcomes**	**№ of participants (studies) Follow-up**	**Certainty of the evidence (GRADE)**	**Relative effect (95% CI)**	**Anticipated absolute effects**	
				**Risk with Placebo**	**Risk difference with Liraglutide**
**GRADE Working Group grades of evidence**<br>**High certainty:** we are very confident that the true effect lies close to that of the estimate of the effect.<br>**Moderate certainty:** we are moderately confident in the effect estimate: the true effect is likely to be close to the estimate of the effect, but there is a possibility that it is substantially different.<br>**Low certainty:** our confidence in the effect estimate is limited: the true effect may be substantially different from the estimate of the effect.<br>**Very low certainty:** we have very little confidence in the effect estimate: the true effect is likely to be substantially different from the estimate of effect.					

Explanations

a. There is a high risk of bias in at least one of the studies for this outcome.
b. High heterogeneity. I2: 90%
c. Not significant effect RR 1.08 and 95%CI 0.92-1.27
d. Moderate heterogeneity. I2: 54%
e. No significant effect RR 2.01 95%CI 0.37 - 11.02
f. High heterogeneity. I2: 99%
g. Significant effect MD -3.35 and moderate range 95%CI -4.65 to -2.05
h. Significant effect and moderate range of 95%CI
i. Moderate heterogeneity. I2: 71%
j. High heterogeneity. I2: 92%
k. High heterogeneity. I2: 100%
l. No significant effect and wide range of 95%CI

Supplemental Figure S18: Publication bias

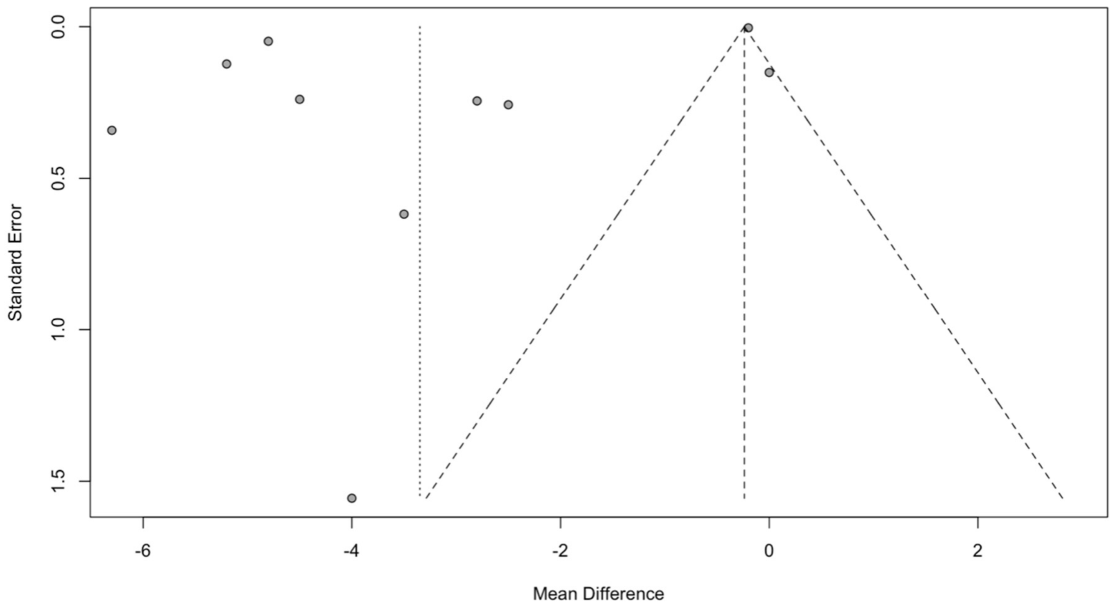

Supplement: Supplementary file 1 [file jcm-11-02998-s001.zip › jcm-1673674-supplementary.pdf]
